# Effects of Eprosartan on Serum Metabolic Parameters in Patients with Essential Hypertension

**DOI:** 10.2174/1874192400701010022

**Published:** 2007-11-14

**Authors:** Evangelos C Rizos, Athanasia Spyrou, Evangelos N Liberopoulos, Eleni C Papavasiliou, Vasilis Saougos, Alexandros D Tselepis, Moses Elisaf

**Affiliations:** 1 Department of Internal Medicine, Medical School, University of Ioannina, 45110 Ioannina, Greece; 2 Laboratory of Biochemistry, Department of Chemistry, University of Ioannina, 45110 Ioannina, Greece

**Keywords:** Eprosartan, hypertension, isoprostane, cholesterol, lipoprotein associated phospholipase A2, glucose

## Abstract

The effect of the anti-hypertensive drug eprosartan on metabolic parameters is currently not extensively documented. We evaluated the effect of eprosartan on parameters involved in atherogenesis, oxidative stress and clotting activity. This open-label unblinded intervention study included 40 adult patients with essential hypertension taking eprosartan. Eprosartan significantly reduced by 8% (p<0.001) the systolic and by 13% (p<.001) the diastolic blood pressure, and in-creased by 24% the time needed to produce oxidative by-products (p=0.001), a marker of oxidative stress. In contrast, ep-rosartan did not alter 8-isoprostane (8-epiPGF2a) levels, another marker of oxidative stress. Additionally, eprosartan re-duced by 14% aspartate aminotransferase and by 21% then alanine aminotransferase activity, while it had a neutral effect on the lipid profile and apolipoprotein levels and did not influence glucose homeostasis, creatinine and uric acid levels. Eprosartan did not affect the clotting/fibrinolytic status (estimated by plasminogen activator inhibitor 1, tissue plasmino-gen activator and a2 antiplasmin levels), or the enzymatic activity of the lipoprotein associated phospholipase A2 (Lp-PLA2) and paraoxonase 1 (PON1). In conclusion, eprosartan should be mainly considered as an anti-hypertensive agent with neutral effects on most of the metabolic parameters in hypertensive patients.

## INTRODUCTION

Eprosartan is an angiotensin II receptor antagonist approved for the treatment of essential hypertension with particular benefit for the secondary prevention of stroke beyond the absolute level of blood pressure reduction [1,2]. However, the effect of eprosartan on various metabolic parameters is currently not extensively documented. Any such effects may be useful, as vascular damage is considered to be a sequel of various underlying mechanisms that are present in hypertensive patients. Some of these mechanisms involve alterations in clotting/fibrinolytic activity, the oxidative damage of vessels and the inflammatory process [3,4]. Moreover, the lipid profile and glucose homeostasis represent additional well-known risk factors for the development of vascular disease in hypertensive patients [5].

Additional actions that could differentiate some of the anti-hypertensive drugs, even within the same class, may affect the evolution of vascular damage. Therefore, we assessed whether eprosartan exerts additional effects on parameters involved in atherogenesis, oxidative stress and clotting activity, beyond its antihypertensive action.

## PATIENTS AND METHODS

### Participants

This open-label intervention study was conducted in patients attending the Metabolism and Lipid Clinic of the University Hospital in Ioannina, Greece. We included adult

patients with essential hypertension defined as systolic blood pressure (SBP) > 140 mmHg and/or diastolic blood pressure (DBP) > 90 mmHg. All individuals were newly diagnosed as hypertensives. Patients with liver or renal disease (serum aminotransferase activity greater than 3-fold (e.g. 120 IU/l), serum creatinine levels > 1.5 mg/dl, hypothyroidism (thyroid stimulating hormone > 5 mIU/ml), heavy alcohol consumption (>3 drinks per day) or patients receiving drugs that could affect lipid metabolism, renal or hepatic function, as well as any drug that could affect the parameters examined in the study were excluded. In particular, the diabetic patients who were on oral anti-diabetic medications did not make any change in the drugs or their doses during the study period.

The Ethical Committee approved the study and all participants gave their formal consent. All patients (n=43) were initially advised regarding lifestyle modification and eprosartan 600 mg once daily was administered in those patients who remained hypertensive after a period of 6 weeks (n=40).

### Measurements

All laboratory determinations were carried out after an overnight fast at the beginning of the study and 12 weeks following eprosartan treatment. Serum levels of total cholesterol (TC), high density lipoprotein cholesterol (HDL-C), triglycerides, glucose, transaminases, creatinine and uric acid were determined by standard methods using an Olympus AU600 Clinical Chemistry analyzer (Olympus Diagnostica, Hamburg, Germany). Serum apolipoprotein (Apo) A1, B and E levels were measured using a Behring Nephelometer BN100 and reagents from Dade Behring Holding GmbH (Liederbach, Germany). Serum insulin levels were measured by an AxSYM insulin assay microparticle enzyme immunoassay on an AxSYM analyzer (Abbott Diagnostics). Fibrinogen, a2-antiplasmin, and platelet activator inhibitor-1 (PAI-1) were measured by Dade Behring BCS analyzer and tissue plasminogen activator (tPA) by ELISA. Low density lipoprotein cholesterol (LDL-C) was calculated by the Friedewald formula [TC - HDL-C - (TRG /5)], insulin sensitivity with the homeostasis model assessment (HOMA) index ([fasting insulin (mU/L) X fasting glucose (mg/dL)] / 405), fractional excretion of uric acid with (urinary uric acid X serum creatinine x 100) / (serum uric acid X urinary creatinine), and body mass index (BMI) as (kg) / height^2^ (m).

For the estimation of oxidative stress we measured the plasma levels of 8-isoprostane (8-epiPGF2a), as well as the susceptibility to oxidation of total serum lipids. The plasma levels of 8-epiPGF2a were determined by means of a competitive ELISA using a commercially available kit (Cayman Chemicals, Ann Arbor, MI), as we recently described [6]. Oxidation of freshly prepared serum was performed at 37ºC in the presence of 100 μM CuSO4. The kinetics of serum oxidation was determined by monitoring the increase in the 245 nm absorbance, every 10 min for 6 h. The lag time, which reflects the resistance of serum lipids to copper-induced oxidation, was calculated as previously described [7,8].

Serum paraoxonase 1 (PON1) activity was determined using paraoxon as a substrate in the presence of 2 mM Ca^+2^ in 100 mM tris-HCL buffer [9]. Lipoprotein-associated phospholipase A_2 _(Lp-PLA_2_) activity in total plasma was determined by the trichloroacetic acid precipitation procedure using 1-0-hexadecyl-2-[^3^H-/sup>H-acetyl]-sn-glycero-3-phosphocholine ([R^3^H]-PAF) (100 μM final concentration) as a substrate [10]. Lp-PLA_2_ activity was expressed as nmol of platelet activating factor (PAF) degraded per min per ml of plasma.

### Statistical Analysis

Descriptive statistics for continuous variables expressed as means with standard deviation (SD), or median with minimum/maximum range were used. Continuous variables were tested for lack of normality with the Kolmogorov–Smirnov test and, accordingly, comparison of the mean values before and after eprosartan treatment was performed with the paired t-test, while comparison of the median values was assessed with the non-parametric Wilcoxon signed rank test.

Statistical analysis was performed using Statistica version 6.0 (StatSoft Inc, Tulsa, Okla). Significance was defined as p<0.05.

## RESULTS

Forty Caucasians (19 females) with essential hypertension were enrolled in the study. Their mean age was 57 years. Most of them were overweight, 17 had metabolic syndrome according to the updated AHA NHLBI statement (defined as 3 or more of the following: waist circumference > 102 cm in men and > 88 cm in women, blood pressure ≥ 130/85 mmHg, triglycerides > 150 mg/dl, HDL-C < 40 mg/dl in men and < 50 mg/dl in women, and fasting glucose ≥ 100 mg/dl), 8 had type 2 diabetes and 18 were smokers (Table 1).

Eprosartan reduced SBP by 8% (p<0.001) and DBP by 13% (p<0.001), while it had a neutral effect on the lipid profile and apolipoprotein levels (Table**2**).

Eprosartan did not affect plasma 8-epiPGF2a levels, whereas it significantly increased by 24% the lag time of total serum oxidation (145 ± 54 min vs 180 ± 58 min, p=0.001) (Table**2**). Eprosartan reduced by 14% the aspartate aminotransferase (form 21 to 18 U/L, p=0.04) and by 21% the alanine aminotransferase (from 24 to 19 U/L, p=0.05) activity (Table**2**).

The administration of eprosartan had no influence on glucose homeostasis, as well as on creatinine and uric acid levels (Table**2**). In addition, eprosartan did not affect clotting or fibrinolytic activity as this was estimated by PAI-1, tPA and a2-antiplasmin (Table**2**). The enzymatic activity of Lp-PLA_2_ and PON1 were not altered significantly following eprosartan treatment (Table**2**).

## DISCUSSION

We found that eprosartan reduced BP levels, but its effects beyond its anti-hypertensive action were limited. Across a wide range of markers related to atherogenesis, such as lipid metabolism, glucose homeostasis, clotting/fibrinolytic process, oxidative stress, and inflammatory mediators for vascular damage, the only additional finding following eprosartan therapy was the reduction in the susceptibility of serum lipids to oxidation as estimated by the increase in lag time, while 8-epiPGF2a, the other marker of oxidative stress, remained unaltered. Thus, any beneficial effect of eprosartan on morbidity/mortality should be mainly attributed to its anti-hypertensive action.

Atherogenesis is considered to be a complex phenomenon implicating vascular oxidative stress, inflammatory pathways and abnormalities in the coagulation/fibrinolysis equilibrium [11]. We estimated the oxidative stress by measurement of 8-epiPGF2a levels, as well as the lag time for the copper-induced oxidation of lipids. 8-epiPGF2a is the most abundant isomer of the F2-isoprostanes, which are isomers of the F2a prostaglandin produced by nonenzymatic peroxidation of arachidonic acid. 8-epiPGF2a is considered to be a marker of oxidative stress, especially of endogenous lipid peroxidation [12-15]. Interestingly, eprosartan increased by 24% the time needed to produce oxidative by-products, while 8-epiPGF2a levels remained unchanged. The

increase of the lag time for LDL oxidation has been previously reported in patients with mild to moderate essential hypertension, and was attributed to the modulation of NADH/NADPH oxidase, which counteracts superoxide production [16].

Eprosartan had no effect on lipid profile, as well as on PON1 and Lp-PLA_2_ that are determinants of lipoprotein function and therefore are involved in atherosclerosis. The neutral effect of eprosartan on lipid profile has been previously reported [17], and was also evident in diabetic patients [18]. PON1 and Lp-PLA_2_ are enzymes related to the inflammatory mechanisms that take part in atherogenesis. PON1 is an esterase that is exclusively associated with HDL in plasma and catalyses the hydrolysis of phospholipid hydroperoxides and cholesteryl ester hydroperoxides, which are formed during LDL oxidation. Thus, PON1 may play an important role in the anti-atherogenic activity of HDL. In contrast, Lp-PLA_2_, an enzyme mainly associated with LDL, is currently related to the inflammatory mechanisms of the atherogenic process. Lp-PLA_2_ exhibits a Ca^+2^-independent phospholipase A_2_ activity and catalyses the hydrolysis of the ester bond at the sn-2 position of the proinflammatory phospholipid mediator PAF. Lp-PLA_2_ also degrades oxidatively fragmented phospholipids, which are formed during the oxidative modification of LDL and are implicated in atherogenesis [19,20]. Neither PON1 nor Lp-PLA_2_ activity were significantly altered after eprosartan therapy, in accordance with the neutral effect of other anti-hypertensive agents on these enzymes [21].

On the other hand, the decrease of transaminase activity following eprosartan administration was consistent with a similar effect following losartan treatment [22]. These findings should be interpreted with caution, because a liver biopsy was not performed in any of our patients to diagnose non-alcoholic fatty liver disease (NAFLD). However, this reduction in liver transaminases may represent a beneficial effect of angiotensin receptor blockers on NAFLD.

Eprosartan had no influence on glucose homeostasis, as was evident from the measurement of glucose, insulin and HOMA index. Consistent with our results, in experimental studies with animals eprosartan failed to induce peroxisome-proliferator–activated receptor (PPAR) γ activity and accordingly failed to demonstrate insulin-sensitizing/antidiabetic effects [23,24]. In humans, eprosartan also has been reported to exert a neutral effect on glucose and insulin levels [17], even in diabetic patients [18].

Eprosartan had a neutral effect on creatinine and uric acid levels, as previously reported [25-27].

Finally, eprosartan did not interfere with the coagulation/fibrinolysis status, although in one report eprosartan seemed to favourably affect these variables [28].

Currently, the anti-hypertensive potency of a drug is considered to be the principal factor that lowers morbidity/mortality and guidelines focus on BP levels as the main target of therapy. In this respect, eprosartan lowered both the SBP and DBP. In contrast, eprosartan had a neutral effect on various metabolic parameters related to the development of vascular disease. The novel findings that eprosartan increased the time needed to produce oxidative by-products and the effect of eprosartan on liver transaminases requires confirmation from larger studies, focusing specifically on eprosartan. This is because there are differences in the properties of the drugs, even in the same class, as was evident from the PPAR γ agonist activity of telmisartan and the uricosuric activity of losartan [29].

In conclusion, based on our findings, eprosartan should be mainly considered as an anti-hypertensive medication with neutral effects on most of the underlying metabolic parameters in hypertensive patients.

## Figures and Tables

**Table 1.** Baseline Characteristics of Study Patients

**N (females/males)**	40 (19/21)
**Age (years)**	57 ± 11
**Weight (kg)**	77 ±15
**BMI (kg/m^2^)**	28.6± 5.2
**Smokers**	18
**Metabolic syndrome**	17
**Diabetes**	8

BMI: body mass index. Mean values ± standard deviation.

**Table 2.** Serum Metabolic Parameters at Baseline and 12 Weeks Following Eprosartan Treatment

	**Baseline**	12 weeks^*^	**P**
**SBP (mmHg) **	154 ± 10	141 ± 12	<0.001
**DBP (mmHg) **	96 ± 8	4 ± 9	<0.001
**TC (mg/dL)**	12 ± 32	219 ± 33	NS
**Triglycerides (mg/dL)**	115 ± 50	113 ± 39	NS
**HDL-C (mg/dL)**	53 ± 8	55 ± 10	NS
**LDL-C (mg/dL)**	134 ± 24	42 ± 28	NS
**ApoA1 (mg/dL)**	145 ± 20	153 ±25	NS
**ApoB (mg/dL)**	86 ± 22	95 ±20	NS
**ApoE (mg/dL)**	3.8 ± 0.7	3.9 ± 1.4	NS
**Lp(a) (mg/dL)**	10 (8-72)	10 (8-11)	NS
**Glucose (mg/dL)**	94 ± 12	96 ± 13	NS
**Insulin (mU/L)**	8.8 ± 3.4	9.9 ± 5.5	NS
**HOMA index**	2.2 ±0.9	2.6 ±1.5	NS
**AST (U/L)**	21 ± 4.6	18 ± 4.6	0.04
**ALT (U/L)**	24 ± 8	19 ± 8	0.05
**Creatinine (mg/dL)**	0.86 ±0.11	0.87 ±0.12	NS
**Uric acid (mg/dL)**	4.5 ±1.5	4.7 ±1.4	NS
**FE uric acid (%)**	13 ±6	10 ± 3	NS
**Fibrinogen (mg/dL)**	343 ±85	347 ± 98	NS
**PAI-1 (U/L)**	3.4 ± 2.6	3.0 ± 1.3	NS
**tPA (ng/mL)**	7.1 ± 3.2	8.6 ± 4.8	NS
**A2-antiplasmin (%)**	100 ± 9	101 ± 17	NS
**8-epiPGF2a (pg/mL)**	61 ± 20	67 ± 24	NS
**Lag time (min)**	145 ± 54	180 ± 58	0.001
**PON1 (U/L)**	60 (20-175)	59 (27-201)	NS
**Lp-PLA_2_ (nmol/mL/min)**	29 ± 8	34 ± 16	NS

SBP: systolic blood pressure; DBP: diastolic blood pressure; TC: total cholesterol; LDL-C: low density lipoprotein cholesterol; HDL-C: high density lipoprotein cholesterol; ApoA1: apolipoprotein A1; ApoB: apolipoprotein B; ApoE: apolipoprotein E; Lp(a): lipoprotein (a); HOMA: homeostasis model assessment index; AST: aspartate aminotransferase; ALT: alanine aminotransferase; FE: fractional excretion; PAI-1: platelet activator inhibitor-1; tPA: tissue plasminogen activator; 8-epiPGF2a: 8-isoprostanes; PON1: paraoxonase 1; Lp-PLA2: lipoprotein-associated phospholipase A2. ^*^Mean values ± standard deviation or median with min/max range. ^**^NS: not significant.
